# Depression Treatment Status of Economically Disadvantaged African American Older Adults

**DOI:** 10.3390/brainsci10030154

**Published:** 2020-03-07

**Authors:** Sharon Cobb, Mohsen Bazargan, Jessica Castro Sandoval, Cheryl Wisseh, Meghan C. Evans, Shervin Assari

**Affiliations:** 1 School of Nursing, Charles R Drew University of Medicine and Science, Los Angeles, CA 90059, USA; sharoncobb1@cdrewu.edu; 2 Department of Family Medicine, Charles R Drew University of Medicine and Science, Los Angeles, CA 90059, USA; Mohsenbazargan@cdrewu.edu (M.B.); cherylwisseh@cdrewu.edu (C.W.); 3 Department of Family Medicine, University of California Los Angeles (UCLA), Los Angeles, CA 90095, USA; meg.e.carlsen@gmail.com; 4 School of Public Health, Charles R Drew University of Medicine and Science, Los Angeles, CA 90059, USA; jessicacastrosandoval@cdrewu.edu; 5 Department of Pharmacy Practice, West Coast University School of Pharmacy, Los Angeles, CA 91606, USA

**Keywords:** depression, African Americans, depressive symptoms, ethnic groups, race

## Abstract

Background: It is known that depression remains largely untreated in underserved communities. Hence, it is desirable to gain more knowledge on the prevalence and correlates of untreated depression among African-American (AA) older adults in economically disadvantaged areas. This knowledge may have the public health benefit of improving detection of AA older adults with depression who are at high risk of not receiving treatment, thereby reducing this health disparity. Objective: To study health and social correlates of untreated depression among AA older adults in economically disadvantaged areas. Methods: Between 2015 and 2018, this cross-sectional survey was conducted in South Los Angeles. Overall, 740 AA older adults who were 55+ years old entered this study. Independent variables were age, gender, living arrangement, insurance type, educational attainment, financial strain, chronic medical conditions, and pain intensity. Untreated depression was the dependent variable. Logistic and polynomial regression models were used to analyze these data. Results: According to the polynomial regression model, factors such as number of chronic medical conditions and pain intensity were higher in individuals with depression, regardless of treatment status. As our binary logistic regression showed, age, education, and number of providers were predictive of receiving treatment for depression. Conclusion: Age, educational attainment, number of providers (as a proxy of access to and use of care) may be useful to detect AA older adults with depression who are at high risk of not receiving treatment. Future research may focus on decomposition of the role of individual-level characteristics and health system-level characteristics that operate as barriers and facilitators to AA older adults receiving treatment for depression.

## 1. Background

Depression is acknowledged as a leading cause of disability and chronic disease burden, and is rapidly increasing among older adults [1]. Even more concerning, this mental health problem is largely untreated for socioeconomically underserved older adults, such as older African Americans (AAs), leading to poor mental and physical health outcomes [1]. It is well established that AAs experience disparities in depression diagnosis and treatment, such as late diagnosis and untreated depressive symptoms [2,3]. However, research over decades has shown depressive symptoms are more prevalent in AA populations [4,5,6]. Furthermore, depression among older AAs tends to be a prolonged, chronic, and severely debilitating condition, leading to decreased daily functioning and health status. Older AAs managing multimorbidity and polypharmacy are more likely to develop depression [7,8]. Findings from the Health Outcome Survey (HOM) that analyzed the prevalence of depression with the Patient Health Questionnaire-2 (PHQ-2) instrument revealed that 16% of older AAs and 9% of non-Hispanic Whites experienced depression [9].

In regard to care, multiple studies have found that older AAs are less likely to receive professional treatment for depression than Whites [10,11,12,13,14,15]. A national population study of Medicaid recipients found that older AAs were more likely to be untreated for depression compared to Whites, Asians, and Hispanic groups [16]. Recent data from the Center for Medicare and Medicaid Services (2017) indicated that the rate of submitted reimbursement claims for depression treatment by health care providers was higher among older non-Hispanic Whites (16%) compared to older AAs (11%). Furthermore, one study found that older AAs are two times more likely than non-Hispanic Whites to receive no pharmacological treatment for depression [16]. Another study (2011) revealed that AAs were less likely than Whites to receive anti-depressant medication following primary care and psychiatry visits. Disparities also exist in regard to non-pharmacological treatment modalities for older AAs as they are less likely to receive counseling, referrals for counseling, or supportive care during primary care visits [17].

Management and treatment of depression can be complex and challenging for older adults, but even more for older AAs, who are less likely to receive care [18]. A higher need for depression treatment exists among those managing multiple chronic health conditions, decreased functional status, and traumatic life events [19]. Treatment of depression in older adults may be difficult due to a higher risk of adverse events, pharmacological interactions, and inappropriate medication use [20]. Untreated depression can result in serious consequences for older adults such as increased disability, poorer self-rated health, and greater pain interference [21]. Older AAs are at an increased risk of loneliness and social isolation than their White counterparts [22]. Older AAs report hopelessness, poor appetite, social isolation, difficulty concentrating, and decreased interpersonal relations as symptoms of depression [23,24], which may differ than the symptoms in Whites [25,26,27,28,29,30,31,32,33,34,35,36,37,38,39,40,41,42,43]. In addition, Hudson and colleagues (2018) revealed that middle-aged AAs identify many barriers to depression treatment, including cultural stigma, mistrust of health care, and being unable to afford treatment [44]. Little is known about depression treatment prevalence, help-seeking behavior, and related factors among underserved older AAs.

Critical Race Theory (CRT) is a useful framework to understand why depression is commonly left untreated in the AA community. CRT suggests that structural racism is more important than individual racism. CRT suggests that racism operates within the societal systems and institutions that are affected and influenced by an oppressive system that systematically excludes minorities. Health care system is one of the many institutions that can be affected by CRT. In the current study, we used CRT to study factors associated with the utilization of depression treatment in the AA community [45]. CRT explains how minority groups are racialized in the society. CRT expands the discourse of minority groups to highlight their lived experiences and the effects of living in a society characterized by structural racism [46]. This type of racism, defined at the macro level, sustains health inequities in many forms, including unequal resources and disproportionate access to medical treatment. Further, it has had major effects on the income, educational attainment, poverty, and health status of older AAs who are historically marginalized [47]. This theory posits that structural racism is more important than individual racism due to its operation within systems, such as health care institutions [47]. This may explain the disparity of AAs receiving less depression treatment than Whites, due to structurally unequal policies and resources, informed by institutional racism. Guided by CRT, this study seeks to understand related factors of untreated depression that are largely affected by structural racism and forms of discrimination among people who have faced racial discrimination throughout their lifetime. The need for depression treatment (both its initiation and its continuity) among older AAs depends in large part on having in place the right institutional policies, the absence of which may otherwise present barriers to obtaining professional treatment [48,49].

## 2. Aims

In a sample of underserved AA older adults in south Los Angeles, the current study was conducted with three aims: (1) to find explanatory factors that were associated with untreated depression (compared to individuals with no depression), (2) to explore social and health factors that were associated with treated depression (compared to individuals with no depression), and (3) to test correlates of treated depression among individuals with depression (factors that distinguish treated from untreated depression).

## 3. Methods

### 3.1. Design and Setting

This study consisted of a cross-sectional survey of low-income AA older adults in South Los Angeles (LA), performed between 2015 and 2018.

### 3.2. Institutional Review Board (IRB)

This study protocol was approved by The Institutional Review Board (IRB) of the Charles R. Drew University of Medicine and Science (CDU), Los Angeles. All participants signed a written informed consent before enrollment in this study and received a financial inventive after enrollment.

### 3.3. Process and Data Collection

Using face-to-face interviews, data was collected on age, gender, insurance type, educational attainment, financial train, living alone, chronic medical conditions (CMCs), pain intensity, depressive symptoms, and previous depression diagnosis and treatment.

### 3.4. Participants

The study recruited AA older adults using convenience sampling in economically disadvantaged areas of South Los Angeles, such as the Compton and Watts areas. Eligibility was limited to older adults who were AA, 55 years or older, could complete an interview in English, and resided in Service Planning Area (SPA) 6. Adults were excluded based on institutionalization, other clinical trial enrollment, or poor cognitive performance, which resulted in 740 AAs adults aged 55+ years.

### 3.5. Measures

The current study collected data on age, gender, educational attainment, financial strain, living alone, CMCs, pain intensity, depressive symptoms, and pervious depression diagnosis and treatment.

#### 3.5.1. Dependent Variable

Untreated depression was defined as self-reported no depression treatment in the presence of a diagnosis of clinical depression or high depressive symptoms. Participants were asked whether they were diagnosed with depression. They were also asked whether they take any medication for their depression. Finally, their depressive symptoms were measured. This study used the 15-item Geriatric Depression Scale (Short Form) (GDS-SF) to evaluate depression [50,51]. Responses were on a “yes” or “no” scale. A summary score was calculated ranging between 0 and 15, with a higher score indicating more depressive symptoms. Excellent reliability and validity has been demonstrated in the GDS-SF, and the measure has been used extensively to assess depression among older adults in both clinical and community settings [50,51]. In the current study, a cut-off of 5.0 is used. This threshold is previously used to classify individuals based on depression using the GDS-SF.

#### 3.5.2. Independent Variables

*Demographic Factors.* Age was an interval variable while gender was treated as dichotomous (1 female, 0 male), and both were covariates in this study.

*Socioeconomic Status (SES).* Covariates also included educational attainment and financial strain. We operationalized educational attainment as an interval variable (years of schooling), and higher scores indicated higher educational attainment. We measured financial strain using three items (α = 0.923) which asked how often the participant did not have enough money for necessities like food, rent/mortgage, clothes, and utility bills. Using a 5-point Likert scale (1 never to 5 always) on each item, we built a sum score of financial strain with a range between 3 and 15, where a higher score reflected more financial strain (lower SES).

*Living Arrangement*. Living arrangements were also operationalized as a dichotomous variable (1 living alone, 0 living with someone else).

*Insurance Type and Providers.* HMO membership was treated as a dichotomous variable (1 yes, 0 no). We also asked participants to report the number of health care providers they see to manage their comorbidities, and this was treated as an interval variable.

*Chronic Medical Conditions (CMCs).* We asked participants whether they had ever been told by a physician that they have any of the following 11 CMCs: hypertension, heart conditions, diabetes, lipid disorder/hypercholesterolemia, cancer, asthma, osteoarthritis, thyroid disorder, chronic obstructive pulmonary disease, rheumatoid arthritis, and gastrointestinal conditions. Even though research demonstrates self-reports are a valid source of CMC information, some measurement bias is also expected [52].

*Pain Intensity.* Using the four subscales of the McGill Pain Questionnaire-Short Form 2 (MPQ-SF-2), we measured pain intensity [53,54,55,56]. This included 22 items asking participants about their experience over the past week of different types of pain and to rate each experience on an 11-point numeric scale (0 none to 10 worst possible). The MPQ-SF-2 subscales include: (a) continuity, (b) intermittence, (c) neuropathic nature, and (d) affective domain. Through averaging the responses to all questions, a total pain score was calculated, where a higher score indicated more pain intensity [53,54,55,56].

## 4. Statistical Note

We used SPSS 22.0 for data analysis. For our univariate analysis, we described our variables in the total sample as well as the following three groups: no depression, depressed but not treated, and depressed and received treatment. We used Analysis of variance (ANOVA) or Chi square test for comparison of all three groups. We used polynomial logistic regression to compare the above three groups with no-depression as the reference group. We then omitted the “no depression” group and ran binary logistic regression to test predictors of treated depression. Odds ratio (OR), 95% confidence interval (CI), and p values were reported.

## 5. Results

### 5.1. Participants

Table 1 (Column 1) reports the characteristics of the study sample. This study included 740 AA individuals who were between the ages of 55 and 96 years (mean, 71.8 ± 8.3). Almost 85% of the participants were 65 years of age or older and 36% of the participants were men. More than 25% of our sample reported not having a high school diploma. Three out of five participants lived alone.

Only 25% of the sample reported receiving their medical care from a Health Maintenance Organization (HMO). Almost 58% of participants had been diagnosed with at least four physical chronic conditions (4.01 ± 1.95). Of the participants, 34% reported being diagnosed with diabetes mellitus, 14% had stroke, and 29% had heart conditions. Participants reported visiting an average of 2.19 (SD = 1.35) health care providers to manage their co-morbidities.

### 5.2. Treated and Untreated Depression

One hundred and thirty-three or 18% of participants indicated that their physician had diagnosed them with depression. However, only 66% of them admitted that they were receiving medical care for their depressive symptoms. In addition, during the data collection of this study, another 69 participants who screened positive with moderate to severe depressive symptoms (using the GDS-SF) had never been diagnosed by their providers for depression. Together, 201 (27%) participants indicated that their providers diagnosed them with depression or, during collection of data, screened positive with moderate to severe depression. However, only 44% (88/201) of these individuals were receiving medical interventions for their depressive symptoms.

### 5.3. Bivariate Analysis

Table 1 (Columns 2–5) presents bivariate associations between depression and other variables. As this table shows, age, educational attainment, living arrangement, financial strain, number of providers, number of physical chronic conditions, and level of pain were all associated with having and being treated for depression. Adults 75 years of age and older were more likely to be left untreated for depressive symptoms compared to middle-aged participants. Participants with no high school diploma, those living alone, and those with a higher level of financial strain also were less likely to receive treatment for their depressive symptoms. Participants with a higher number of physical chronic conditions and a higher level of pain were at a higher risk of suffering from depression and had a higher chance of receiving medical intervention for depression than their counterparts with a lower level of pain and a lower number of physical chronic conditions.

### 5.4. Multinomial Logistic Regression

Table 2 shows the summary of the results of a multinomial regression model with a three-level outcome: no depression, untreated depression, and treated depression. Age, educational attainment, financial strain, number of providers, level of pain, and number of chronic conditions were associated with untreated or treated depression.

Comparing the middle-aged and the oldest (75 years and older) participants, middle-aged participants had a higher probability of belonging to the untreated group of survey respondents. The odds of untreated depression increased 2.471 times (95% CI: 1.187–5.146) for middle aged participants (55–64), compared with participants with no depression. Similarly, respondents with no high school diploma were 0.542 times (95% CI: 0.591–0.904) less likely to be untreated for depression, compared with participants who have a high school diploma. (Table 2)

Controlling for all other variables, a higher level of financial strain increased the odds of having depression but being untreated for it by 1.369 (1/0.731 = 1.369 95% CI: 0.591–0.904) times, compared with those with no depression. However, financial strain was not associated with having depression and receiving medical intervention for depression, compared with those with no depression (OR: 0.898; 95% CI: 0.693–1.164).

Table 2 shows that, controlling for all other relevant variables, the odds of receiving medical intervention for depression increases 1.247 times (95% CI: 1.049–1.483) when the participant has more providers, compared with those with no depression. However, the number of providers was not different between those with untreated depression and those with no depression (OR: 0.881; 95% CI: 0.721–1.076).

Participants suffering from a higher number of chronic conditions were more likely to have depression but not receive medical treatment for it (OR: 1.188; 95% CI: 1.035–1.363), or to have depression and receive medical treatment for it (OR: 1.348; 95% CI: 1.154–1.576), compared with those with no depression. However, the odds ratio was stronger for being under care than for not being under care for depression (OR: 1.348 vs. 1.188).

Similarly, a higher level of pain was associated with higher odds of having depression but not receiving medical treatment (OR: 1.278; 95% CI: 1.132–1.442) or having depression and receiving medical treatment (OR: 1.219; 95% CI: 1.089–1.363) when compared with no depression.

### 5.5. Multivariate Binary Logistic Regression

Table 3 presents the results of a logistic regression model comparing untreated and treated depression. This model was tested using only 201 participants who were diagnosed by providers or who screened positive with the GDS-SF. Only three variables (age, educational attainment and number of providers) had association significant enough to differentiate untreated from treated depression. Participants with a high school diploma had 2.52 (95% CI: 1.193–5.322) odds of receiving medical intervention for depression, compared with their counterparts with no high school diploma. In addition, participants aged 75 and older had 0.432 (95% CI: 0.191–0.974) less odds of being treated for their depression, compared with participants who were 65–74 years old. Finally, participants who reported a higher number of providers had higher odds of receiving a medical intervention for their depression. For each additional provider, the odds of receiving care increased by 1.456 (95% CI: 1.145–1.875) times. Controlling for all other relevant variables, the number of physical chronic conditions and level of pain were no longer associated with receiving medical interventions for depression.

## 6. Discussion

This study showed that among older AAs, an estimated 33% are not receiving treatment for their depression. Furthermore, individuals who reported multiple chronic conditions, more health care providers, and a higher level of pain were more likely to have depression care. Untreated depression was associated with belonging to the oldest age cohort (75 years and older) and lower educational attainment (less than a high school diploma). This study is consistent with previous findings indicating that AAs experience lower rates of depression and mental health treatment, as well as decreased access to and quality of depression care [57,58,59,60,61].

As suggested by Critical Race Theory (CRT), this finding is linked to the systemic structural underpinning of mental health treatment barriers to depression among AA populations [45,46]. On an institutional level, treatment disparities for older AAs are linked to structural racism and discrimination, cultural mistrust, provider stereotypes and bias [62]. Even though older AAs who have a larger network of health care providers and more chronic conditions are more likely to receive depression treatment, the effects of racial disparities continue to persist. In 2010, Osborn and colleagues showed that older AAs with diabetes and depression had a lower rate of anti-depressant use compared to their White counterparts [13]. This disparity also exists among primary care and psychiatry health care providers who primarily serve non-White patients [14]. This racial inequity may be discouraging for older AAs who may find it difficult to obtain depression treatment from health care providers close to where they live.

Williams and colleagues found that only 45% of AAs with depression had received any form of treatment during their lifetime [63]. Assari showed that clinical depression is associated with higher levels of depressive symptoms in AAs than Whites [64]. In another study, he showed that depression is associated with more financial needs in AAs than Whites [25]. Blazer et al. also showed that depression among AAs is more severe and disabling than among Whites [62].

Thus, having access to higher number of providers is crucial for depression treatment. Similarly, certain conditions such as multiple chronic ailments and pain seem to push AA older adults toward treatment. This might be because of high levels of stigma associated with depression in the AA community [65,66]. We argue that pain and chronic medical conditions may be the primary reason AA older adults seek medical care, mainly in primary care settings. Some providers may then detect or evaluate symptoms of depression and provide treatment.

Moreover, AAs who receive mental health services view their care less favorably compared to Whites [67], suggesting that they may encounter the lingering effects of structural racism within health care settings. Related sociocultural factors, such as race-based and cultural stigma, can affect perceptions of their treatment [65,66]. In 2012, Jimenez and colleagues revealed that older AAs had differing beliefs on the causes of mental problems compared to Whites, which can affect treatment decision making [68]. Religiosity and spirituality can influence treatment decisions as this population may rely on faith-based practices as a coping mechanism for depression. The population may participate in culturally-based coping behaviors, such as religious practices, and avoid professional treatment for depression [10]. AA’s health risk behaviors may be indirect effects of structural factors, as posited by CRT. Treatment for depression provided by health care providers should involve consideration of sociocultural values and preferences, in addition to addressing the manifestations of structural racism rooted within specific health care settings [23,46,69].

The results are important because if depression is present, lack of treatment has the potential to jeopardize the participant’s health status. Failure to detect and treat depression is a risk factor of a wide range of health problems such as drug use, aggression, suicide, chronic disease, suicide, and mortality. In the absence of screening by health care providers, depression may be going undetected. Untreated depression results in worse health outcomes.

Additionally, we found that older AAs who were managing multiple chronic conditions and those who had more health care providers were more likely to be receiving treatment for depression. In 2014, Agyemang and colleagues found that AAs diagnosed with depression, comorbid diabetes, and hypertension were more likely to receive depression treatment as opposed to those diagnosed with depression alone [70]. Managing multiple conditions may necessitate frequent health care visits which can prove beneficial, since providers can routinely assess mental health status and provide timely treatment. Yet due to the myriad of patient, provider, and treatment factors, AAs may face competing clinical priorities of comorbid health conditions and experience a lack of comprehensive primary care services, resulting in inadequate treatment of their depression and poorer health outcomes [3,71,72]. Existing barriers, such as low access to mental health services by any individual regardless of minority group or age, are exacerbated by a lack of resources, such as unhurried patient–provider interactions [73]. It is imperative that AAs have a supportive collaboration with providers for optimal management of their health status, including depression and comorbidity management, which may explain our findings [74].

The findings of this study also revealed that older AAs with poor self-rated health have a higher likelihood of depression treatment. Previous studies also found that individuals with perceived poor health, including those reporting a cluster of depressive symptoms, are more likely to take advantage of mental health services [75,76]. African Americans experience poorer self-rated health compared to other groups, including Whites, an example of racial disparities. However, this study suggests there is a complex relationship between self-rated health and depression treatment, and thus demonstrates the significance of perceived health in understanding treatment decision making.

Adults between the ages of 50 and 64 years and ages 75 years and older were also less likely to have treated depression. One study found that referrals for depression treatment were fewer for adults 75 years and older compared to other age cohorts [77]. In 2016, Choi and colleagues found that in 62,763 ambulatory and primary care visits, treatment for depression was not provided in over 30% of visits by older depressed adults [78]. Providers are not paying adequate attention to depression in the oldest population, and so their patients may not be getting adequate treatment or may not be experiencing improvement in depressive symptoms following treatment [79]. This group is at a high risk of polypharmacy and medication interaction, which, coupled with the management of multiple chronic conditions, may lead to untreated depression [8,80]. However, further study is needed to understand the factors contributing to untreated depression among the younger-old cohort (ages 50–64 years).

With respect to educational attainment, studies show that individuals with lower educational levels were more likely to have untreated depression [19]. It is recognized that higher educational attainment may be beneficial in ensuring older adults receive depression treatment and are adherent with medication [81,82]. Future studies should examine the effect of educational attainment on depression treatment (comparing AAs and Whites) to see if disparities are observed.

Financial strain was also found to be associated with untreated depression, which is not unsurprising among this population. In 2016, Grace et al. found mental health service use was lower among AAs, who reported greater stressful life events and financial strain, compared to Whites [83]. Szanton and colleagues reported that financially strained older AAs who received managed depression care experienced a decrease in depressive symptoms [84]. Another study reported that financial strain is a significant predictor of clinical depression on follow up among older adults being treated for depression [85]. Additionally, research indicates socioeconomic inequalities in the incidence of depression widen into older adulthood, suggesting that older adults with more financial strain and lower educational attainment are at increased risk of developing depressive symptoms [86]. Future work needs to focus on the underlying mechanisms that link financial strain and depression treatment outcomes.

Both pharmacological and alternative therapies should be utilized for this population, especially for individuals with comorbidities [87]. AAs who receive outpatient mental health care incur lower costs for inpatient and emergency medical care [88]. Yet AAs are more likely to be non-adherent to anti-depressant medication [89]. Harman, Edlund, and Fortney found that compared to Whites, AAs are more likely to receive psychotherapy but less likely to fill their anti-depressant medication [90]. Literature shows that older AAs have difficulty filling their medications due to low access to pharmacy services, lack of medication knowledge, and cost. AAs differ in their presentation of depression and other related mental health problems and may exhibit symptoms resembling a psychotic disorder, leading to misdiagnosis and inappropriate treatment [66]. Clinicians should exercise caution when evaluating treatment options for depression and should assist these individuals with medication supply and adherence.

There is growing evidence that a combination of anti-depressant therapy and non-pharmacological treatment may be beneficial for both the physical and mental health of older adults [20]. Fuentes and Aranda found that older AAs are more likely to benefit from collaborative and integrated care for depression treatment [69]. Another study by Arean et al., in 2005, showed that a collaborative care model in primary care significantly improves the rate of depression among older minorities, including AAs [69,73]. Faith-based organizations providing depression services also saw increased rates of AAs obtaining care compared to Whites and U.S.-born Hispanics [91]. The application of a collaborative care model for older minority adults may improve the quality of mental health care and access to resources in primary care, which should include both pharmacological treatment and managed counseling support [73,92]. Patient navigation programs that include these components may lead to greater willingness to receive treatment.

### Limitations

This study had a few methodological limitations that were inherent to the study design. Being a cross-sectional study, causal inferences were not possible. There may be existing self-report bias. We did not cross-validate self-reported data with diagnoses in medical records. We did not include other related mental health problems such as anxiety or substance use in our analyses. Substance use may partially explain the differences between those who do and do not receive treatment. It is important to note that depression in this study was not diagnosed by a psychiatrist. Instead, participants were categorized as depressed if they self-reported their depression diagnosis or screened positive when they completed the GDS-SF. Finally, non-random sampling decreases the generalizability of the results. Despite these limitations, the findings further the literature on factors associated with depression treatment among older AAs in low-income urban settings.

## 7. Conclusions

Among older AAs with depression residing in underserved areas, a higher number of chronic health conditions, health care providers, level of pain, and educational attainment may shape the chance of receiving treatment. Knowledge regarding these factors may help policy makers and clinicians to identify those at risk for untreated depression and to provide culturally sensitive care, including pharmacological aids (e.g., anti-depressant medications) and relevant patient and provider education. There is also a need to develop collaborative care programs that include patient navigation, resources, and guided support.

## Figures and Tables

**Table 1:** Descriptive statistics and bivariate correlations between depression and independent variables (*n* = 740).

Independent Variables	Total	Depression			*p*
		No *n* (%)	Untreated *n* (%)	Treated *n* (%)	
	*n* (%)	*n* (%)	*n* (%)	*n* (%)	
**Gender**					
Male	266 (36)	190 (71)	46 (17)	30 (11)	0.513
Female	473 (64)	348 (74)	67 (14)	58 (12)	
**Age**					
55–64 (Young–Old)	109 (15)	**59 (54)**	**24 (22)**	**26 (24)**	**0.000**
65–74 (Mid–Old)	360 (49)	**269 (74)**	**54 (15)**	**37 (10)**	
≥75 (Old–Old)	260 (36)	**210 (81)**	**25 (10)**	**25 (10)**	
**Educational Attainment**					
No High School Diploma	183 (25)	**123 (67)**	**39 (21)**	**21 (11)**	**0.033**
High School Diploma	556 (75)	**415 (75)**	**74 (13)**	**67 (12)**	
**Lived Alone**					
No	294 (40)	**231 (79)**	**35 (12)**	**28 (10)**	**0.016**
Yes	445 (60)	**307 (69)**	**78 (13)**	**60 (13)**	
**HMO Membership**					
No	477 (65)	348 (73)	70 (15)	59 (12)	0.749
Yes	262 (35)	190 (72)	43 (16)	29 (11)	
	**Mean (SD)**	**Mean (SD)**	**Mean (SD)**	**Mean (SD)**	
**Financial Strains (1–5)**	4.17 ± 1.13	**4.36 ± 1.01**	**4.75 ± 1.24**	**3.68 ± 1.29**	**0.000**
**Number of Providers (1–10)**	2.19 ± 1.35	**2.12 ± 1,25**	**2.01 ± 1.29**	**2.85 ± 1.82**	**0.000**
**# of Major Chronic Conditions (1–11)**	4.01 ± 1.95	**3.68 ± 1.81**	**4.75 ± 1.96**	**5.50 ± 1.86**	**0.000**
**Level of Pain**	2.03 ± 2.25	**1.49 ± 1.76**	**3.22 ± 2.58**	**3.80 ± 2.94**	**0.000**

Bold numbers are statistically significant.

**Table 2:** Multinomial logistic regression on factors associated with treated depression and depressed but with no medical care, compared to no depression.

Independent Variables	Untreated Depression			Treated Depression		
	OR	95% CI	*p*	OR	95% CI	*p*
**Gender**						
Male	0.856	0.521–1.407	0.541	0.831	0.469–1.474	0.527
Female	1.00					
**Age**						
55–64	**2.471**	**1.187–5.146**	**0.016**	2.189	0.975–4.915	0.058
65–74	1.756	0.988–3.092	0.051	0.917	0.499–1.687	0.782
≥75	**1.00**					
**Educational Attainment**						
No High School Diploma	**0.542**	**0.326–0.902**	**0.018**	1.291	0.678–2.456	0.437
High School Diploma	**1.00**					
**Live Alone**						
No	1.453	0.882–2.395	0.142	1.129	0.642–1.984	0.673
Yes	1.00			1.00		
**Financial Strain**	**0.731**	**0.591–0.904**	**0.004**	0.898	0.693–1.164	0.416
**HMO Membership**						
No	1.258	0.769–2.056	0.361	0.934	0.531–1.641	0.812
Yes	1.00			1.00		
**# of Providers**	0.881	0.721–1.076	0.213	**1.247**	**1.049–1.483**	**0.012**
**# of Chronic Conditions**	**1.188**	**1.035–1.363**	**0.015**	**1.348**	**1.154–1.576**	**0.000**
**Level of Pain**	**1.219**	**1.089–1.363**	**0.001**	**1.278**	**1.132–1.442**	**0.000**

Bold numbers are statistically significant. Reference Category: No Depression; −12 Log Likelihood: 875.6; *df*: 20; p < 0.001; Nagelkerke *R* Square: 0.271.

**Table 3:** Logistic regression on distinction of untreated and treated depression among individuals with depression (*n* = 221).

Independent Variables	Treated Depression		
	OR	95% CI	*p*
**Gender**			
Female	0.915	0.453–1.847	0.805
Male	1.00		
**Age**			
≥75	0.876	0.338–2.270	0.786
65–74	**0.432**	**0.191–0.974**	**0.043**
55–64	1.00		
**Educational Attainment**			
High School Diploma	**2.520**	**1.193–5.322**	**0.015**
No High School Diploma	**1.00**		
**Live Alone**			
Yes	0.613	0.296–1.269	0.187
No	1.00		
**Financial Strain**	1.206	0.887–1.638	0.231
**HMO Membership**			
Yes	0.729	0.365–1.457	0.371
No	1.00		
**# of Providers**	**1.465**	**1.145–1.875**	**0.002**
**# of Chronic Conditions**	1.073	0.886–1.298	0.471
**Level of Pain**	1.088	0.949–1.247	0.229

Bold numbers are statistically significant.
